# Resveratrol Induces Premature Senescence in Lung Cancer Cells via ROS-Mediated DNA Damage

**DOI:** 10.1371/journal.pone.0060065

**Published:** 2013-03-22

**Authors:** Hongmei Luo, Aimin Yang, Bradley A. Schulte, Michael J. Wargovich, Gavin Y. Wang

**Affiliations:** 1 Department of Pathology and Laboratory Medicine, Medical University of South Carolina, Charleston, South Carolina, United States of America; 2 Cell and Molecular Pharmacology and Experimental Therapeutics, Medical University of South Carolina, Charleston, South Carolina, United States of America; Wayne State University School of Medicine, United States of America

## Abstract

Resveratrol (RV) is a natural component of red wine and grapes that has been shown to be a potential chemopreventive and anticancer agent. However, the molecular mechanisms underlying RV's anticancer and chemopreventive effects are incompletely understood. Here we show that RV treatment inhibits the clonogenic growth of non-small cell lung cancer (NSCLC) cells in a dose-dependent manner. Interestingly, the tumor-suppressive effect of low dose RV was not associated with any significant changes in the expression of cleaved PARP and activated caspase-3, suggesting that low dose RV treatment may suppress tumor cell growth via an apoptosis-independent mechanism. Subsequent studies reveal that low dose RV treatment induces a significant increase in senescence-associated β–galactosidase (SA-β-gal) staining and elevated expression of p53 and p21 in NSCLC cells. Furthermore, we show that RV-induced suppression of lung cancer cell growth is associated with a decrease in the expression of EF1A. These results suggest that RV may exert its anticancer and chemopreventive effects through the induction of premature senescence. Mechanistically, RV-induced premature senescence correlates with increased DNA double strand breaks (DSBs) and reactive oxygen species (ROS) production in lung cancer cells. Inhibition of ROS production by N-acetylcysteine (NAC) attenuates RV-induced DNA DSBs and premature senescence. Furthermore, we show that RV treatment markedly induces NAPDH oxidase-5 (Nox5) expression in both A549 and H460 cells, suggesting that RV may increase ROS generation in lung cancer cells through upregulating Nox5 expression. Together, these findings demonstrate that low dose RV treatment inhibits lung cancer cell growth via a previously unappreciated mechanism, namely the induction of premature senescence through ROS-mediated DNA damage.

## Introduction

Lung cancer is responsible for more cancer deaths in the United States than the combined mortality of colorectal, breast and prostate cancer [1]. Even with the newer advanced therapeutic approaches, the 5-year overall survival rate is less than 16% and has not changed appreciably over many decades [1], [2]. This poor prognosis emphasizes the urgent need for the development of novel strategies for the prevention and more effective treatment of this deadly disease. Natural products (NPs) are widely used by Americans as complementary and alternative medications (CAM) for the prevention and treatment of various human diseases including cancers [3], [4]. The use of NPs as antitumor agents for the management of human cancers is an attractive idea because they are readily available and exhibit little or no toxicity [3], [5]–[7]. Resveratrol (RV) is one of such NPs and has been shown to exhibit both anticancer and chemopreventive potentials [3], [8]–[10]. However, the exact molecular mechanisms underlying RV's chemopreventive and anticancer effects are not completely understood. The goal of this study was to define the role of premature senescence in RV-induced antitumor effects in lung cancer cells.

Cellular senescence is a state of permanent cell cycle arrest that can be triggered by a variety of stresses including DNA damage, telomere shortening and oxidative stress [11]–[13]. The two major categories of cellular senescence are replicative senescence and stress-induced premature senescence (SIPS). Replicative senescence was first described by Hayflick and Moorhead in human fibroblasts after cells underwent extensive replication as a consequence of serial culture passages [14]. Subsequently, it was found that cells also can undergo SIPS in response to DNA-damaging agents such as ionizing radiation and anticancer chemotherapeutics [11]–[13], [15]. Cells undergoing SIPS are morphologically indistinguishable from replicatively senescent cells and exhibit many of the characteristics ascribed to replicative senescence, such as increased senescence associated β-galactosidase (SA-β-gal) activity and increased p53 and p21 expression [11]–[13], [15]–[17]. Although telomere shortening was thought to be the major cause for replicative senescence, premature senescence can occur in a telomerase- and telomere shortening-independent mechanism [18], [19]. Senescence limits the life span and proliferative capacity of cells, therefore the induction of senescence is regarded as an important mechanism of cancer prevention [20]–[22]. More importantly, emerging evidence has demonstrated that therapy-induced senescence is a critical mechanism through which many anticancer agents inhibit the growth of tumor cells [11], [12], [23]. Interestingly, it has been shown that therapy-induced senescence can be achieved at much lower doses of chemotherapy than those required to induce apoptosis, indicating that high doses of anticancer agent may cause apoptosis whereas low level treatments primarily induce senescence in cancer cells [12]. Compared to the traditional apoptosis inducing strategies, this low dose approach can significantly reduce the side effects of anticancer therapy and thus improve the quality of life for cancer patients. Therefore, it is important to understand whether low dose RV can suppress lung cancer cell growth via the induction of premature senescence.

RV is a nontoxic natural polyphenolic compound found in abundance in grapes, red wines, mulberries and other edible plants. Recent clinical trials have indicated that RV is well tolerated and relatively safe for use in humans [5]–[7]. RV has been shown to inhibit the proliferation of a variety of cancer cells via inactivation of various cell survival pathways including the PI3-kinase/AKT pathway [24]. RV also has been shown to exhibit potential chemopreventive activity in several carcinogen-induced tumor models including breast, intestine, liver, esophagus and colon [3], [25], [26]. Moreover, it has been reported that RV treatment inhibits pancreatic cancer growth and enhances the anticancer effects of gemcitabine possibly via suppression of NF-КB activation and down-regulating the expression of cyclin D1, COX-2, ICAM-1, MMP-9 and survivin in tumor tissues [27]. Although previous studies have indicated that RV, at high doses, can inhibit the proliferation of cancer cells by inducing apoptosis [28]–[31], a major challenge for the use of this CAM is that the concentration of RV required to induce apoptosis in tumor cells *in vitro* is too great to achieve *in vivo* in a clinical setting [5]–[7], [32]. Given that induction of senescence requires a much lower concentration of anticancer agents and thus produces fewer unwanted side effects [12], we sought to investigate if low dose RV treatment could inhibit the growth of cancer cells through the induction of premature senescence. In the present study, we show that low dose RV treatment leads to a significant increase in senescence-associated β–galactosidase (SA-β-gal) staining and elevated p53 and p21 expression in NSCLC cells, suggesting that the anticancer effect of RV is largely attributable to the induction of senescence in lung cancer cells. Mechanistic studies reveal that RV-induced senescence is associated with increased DNA DSBs and ROS production in lung cancer cells. Moreover, our data also show that inhibition of ROS production by NAC attenuates RV-induced DNA DSBs and premature senescence. Altogether, these findings demonstrate that low dose RV treatment causes premature senescence in lung cancer cells via ROS-mediated DNA damage, which highlight a significant contribution of senescence induction to RV's anticancer effects.

## Results

### RV inhibits the growth of lung cancer cells in a dose-dependent manner

**Figure 1:**
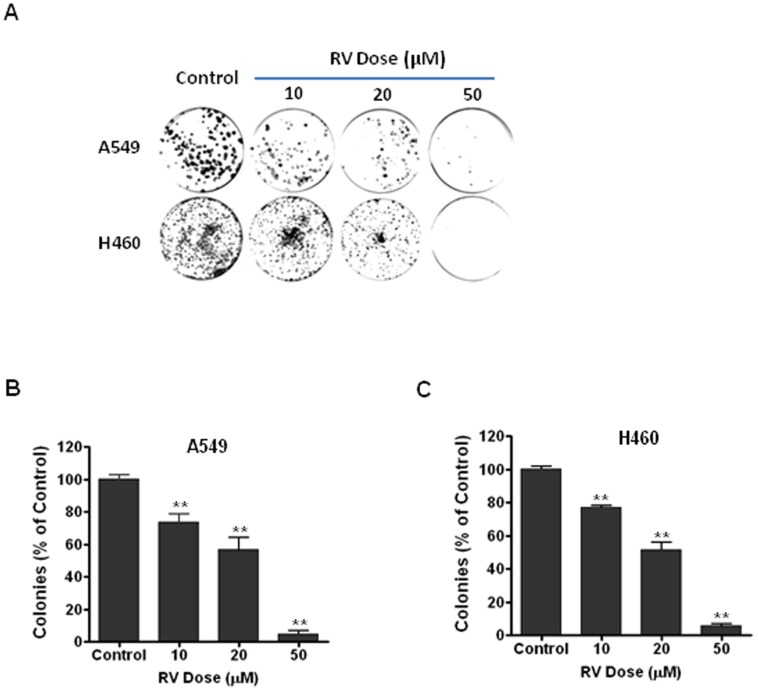
RV inhibits the growth of NSCLC cells in a dose-dependent manner. (**A**) Clonogenic survival assays show that the number of cancer cell-derived colonies decreases with RV dose. (**B**) The results of clonogenic assays were normalized to the clonogenic survival of control A549 cells and are expressed as % of control. (**C**) The results of clonogenic assays were normalized to the clonogenic survival of control H460 cells and are expressed as % of control. **, *p*<0.01 vs. control.

Previous studies have indicated that higher doses of RV treatment may inhibit the proliferation of tumor cells by inducing apoptosis [28]–[31], but a major challenge for this apoptosis-causing strategy is that the concentration required to induce apoptosis in tumor cells *in vitro* is not reachable *in vivo* [5]–[7], [32]. Therefore, it is important to determine if low dose RV treatment affects the growth of tumor cells. To this end, we treated A549 and H460 lung cancer cells with different low doses of RV (0–50 µM) to examine if RV treatment has any impact on the colony formation of NSCLC cells. Clonogenic survival assays demonstrated that even as low as 10 µM of RV treatment can significantly suppress the colony-forming activity of A549 and H460 cells (**Figs. 1A, 1B and 1C**). The data also show that RV-induced suppression of colony formation correlates well with the concentrations of RV, suggesting that RV treatment inhibits the clonogenic growth of NSCLC cells in a dose-dependent manner.

### Low dose RV inhibits lung cancer cell growth via an apoptosis-independent mechanism

**Figure 2:**
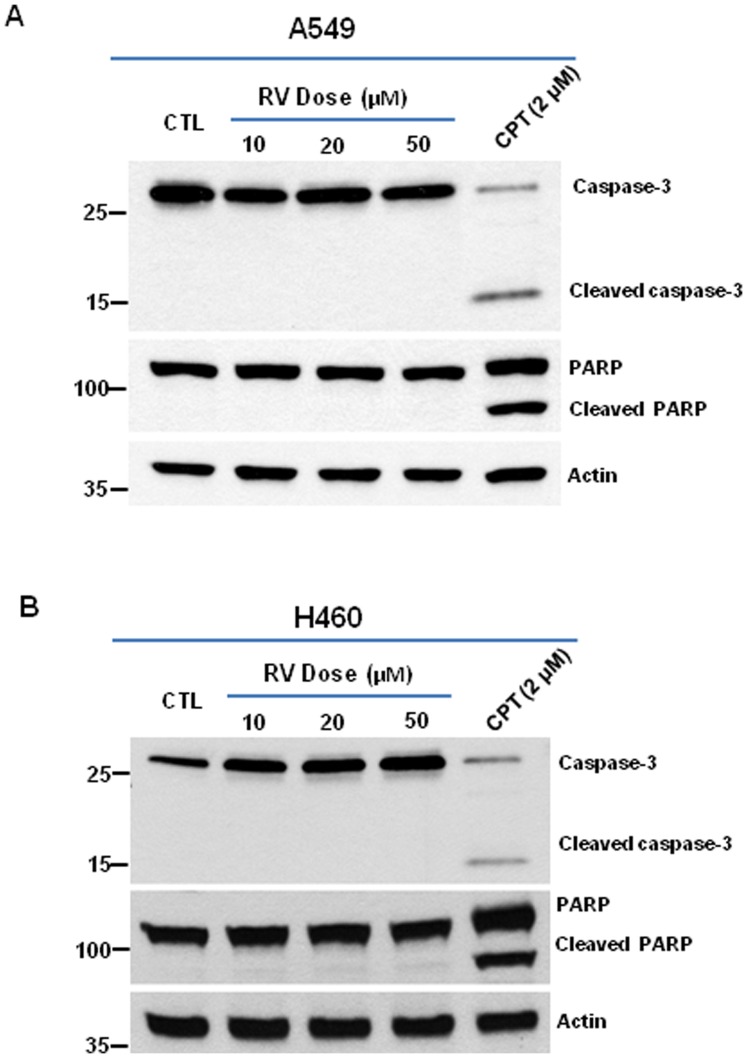
Low dose RV suppresses lung cancer cell growth via an apoptosis-independent mechanism. (**A**) Western blot assays were performed to determine the expression of activated caspase-3 and cleaved PARP in A549 cells. Actin was used as a loading control. (**B**) Western blot assays were performed to determine the expression of activated caspase-3 and cleaved PARP in H460 cells. Actin was used as a loading control.

Although it has been shown that higher doses (100–200 µM) of RV treatment may induce apoptosis in tumor cells [28]–[31], it was unknown if low dose RV suppresses the growth of lung cancer cells through the induction of apoptosis. Because activated caspase-3 and cleaved PARP are well-documented measurements of apoptosis [33], [34], we investigated if low dose RV treatment has any impact on the expression of activated caspase-3 and cleaved PARP in A549 and H460 cells. As shown in **Figure 2**, Western blotting data revealed that low dose RV treatment did not cause any significant changes in the expression of cleaved PARP and activated caspase-3 in either A549 or H460 cells. In contrast, camptothecin (CPT) treatment resulted in a pronounced increase in cleaved PARP and activated caspase-3 expression in both A549 and H460 cells (**Figs. 2A and 2B**). These results strongly suggest that low dose RV inhibits lung cancer cell growth via an apoptosis-independent mechanism.

### RV induces premature senescence in lung cancer cells

**Figure 3:**
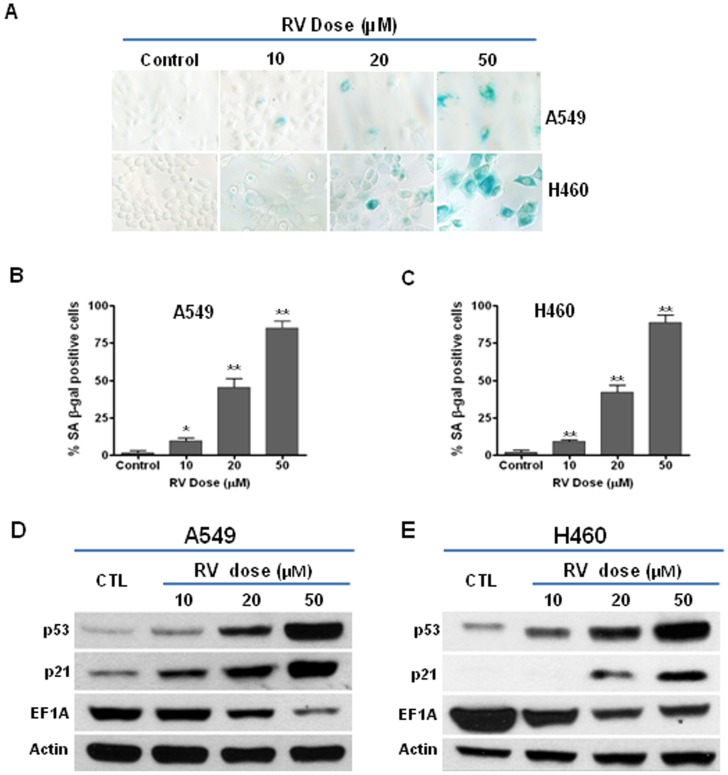
RV induces premature senescence in NSCLC cells. (**A**) SA-β-gal staining increased with RV doses in both A549 cells (upper panel) and H460 cells (lower panel). (**B**) The percentage of SA-β-gal positive senescent cells in RV-treated and control A549 cells is presented as mean ± SEM. (**C**) The percentage of SA-β-gal positive senescent cells in RV-treated and control H460 cells is presented as mean ± SEM. (**D**) Western blot assays were performed to determine the expression of p53, p21 and EF1A in A549 cells. Actin was used as a loading control. (**E**) Western blot assays were performed to determine the expression of p53, p21 and EF1A in H460 cells. Actin was used as a loading control. *, *p*<0.05 vs. control; **, *p*<0.001 vs. control.

It has been proposed that the induction of premature senescence is an important mechanism by which ionizing radiation and many chemotherapeutic agents exert their anticancer effects [11]–[13], [15], [17], [23]. Thus, we sought to examine if low dose RV treatment induces premature senescence in NSCLC cells. Because increased SA-β-gal activity is a well-established biomarker of senescence [16], we investigated if low dose RV treatment induces premature senescence in A549 and H460 cells by SA-β-gal staining. As shown in **Figure 3A**, the results indicate that the number of SA-β-gal positive senescent cells is markedly increased in RV-treated versus control A549 and H460 cells. Moreover, the percentage of SA-β-gal positive cells increases with the dose of RV, suggesting that RV treatment induces premature senescence in lung cancer cells in a dose-dependent manner (**Figs. 3B and 3C**).

We also examined the expression levels of p53 and p21, two important molecules involved in the regulation of senescence [12], [15], [17], [35], in RV-treated NSCLC cells. Western blotting data demonstrated that the expression levels of p53 and p21 were significantly increased in RV-treated cells, compared with control A549 and H460 cells (**Figs. 3D and 3E**). These results suggest that the p53–p21 pathway is involved in RV-induced premature senescence in lung cancer cells. Interestingly, our data also show that RV treatment significantly down-regulates the expression of EF1A in A549 and H460 cells (**Figs. 3D and 3E**), suggesting that EF1A may play an important role in regulating RV-induced premature senescence in NSCLC cells.

### RV treatment causes DNA damage and increases ROS production in lung cancer cells

**Figure 4:**
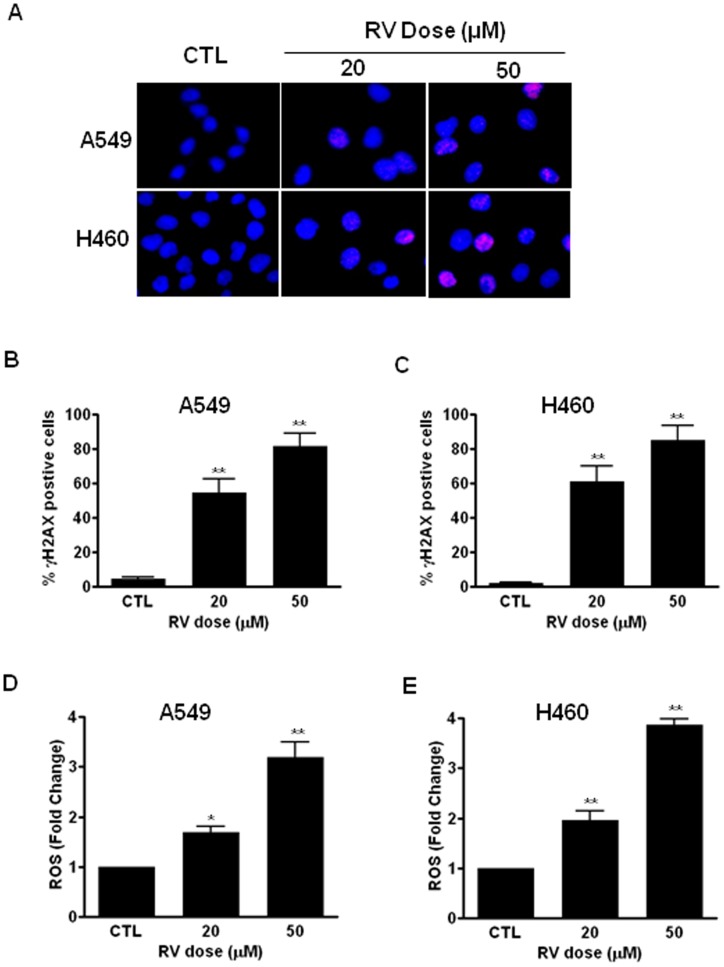
RV treatment leads to DNA damage and increases ROS production in NSCLC cells. (**A**) DNA DSBs were determined by γH2AX immunofluorescent microscopy as previously described (16). Representative immunofluorescent images of γH2AX foci in RV-treated A549 and H460 cells are presented. (**B**) The percentage of γH2AX foci positive cells in RV-treated A549 cells is presented as mean ± SEM. (**C**) The percentage of γH2AX foci positive cells in RV-treated H460 cells is presented as mean ± SEM. (**D**) The levels of ROS were measured by DCF-DA staining and flow cytometric analyses at 24 h after RV treatment. The levels of ROS in RV-treated A549 cells are presented as fold change compared to the levels in control cells. (**E**) The levels of ROS in RV-treated H460 cells are presented as fold change compared to the levels in control cells. *, *p*<0.05 vs. control; **, *p*<0.01 vs. control.

Many chemotherapeutic agents and radiation kill tumor cells through the induction of DNA damage. Phosphorylated H2AX (γH2AX) is a robust marker of DNA DSBs [36]. To determine if DNA damage contributes to RV-induced anticancer effects, we performed γH2AX foci assays to examine if RV treatment causes DNA DSBs in lung cancer cells. As shown in **Figure 4**, our data demonstrate that RV treatment results in a significant increase in the formation of γH2AX foci in both A549 and H460 cells (**Figs. 4A, 4B and 4C**). As the formation of γH2AX foci is an important surrogate of DNA DSBs [**36**], these results demonstrate for the first time that the anticancer effect of RV is attributable at least in part to RV-induced DNA damage in NSCLC cells.

We and others have demonstrated that ROS play a critical role in mediating genotoxic stress-induced DNA damage [37], [38]. Therefore, we hypothesized that RV may cause DNA DSBs via increased ROS production in NSCLC cells. To test this hypothesis, we investigated if RV treatment has any impact on ROS production in lung cancer cells. DCF-DA staining and flow cytometric assays showed that the levels of ROS were markedly increased in RV-treated A549 and H460 cells compared with that of control cells (**Figs. 4D and 4E**). These results suggest that RV may induce lung cancer cell premature senescence via ROS-mediated DNA damage.

### NAC attenuates RV-induced DNA damage and premature senescence in lung cancer cells

**Figure 5:**
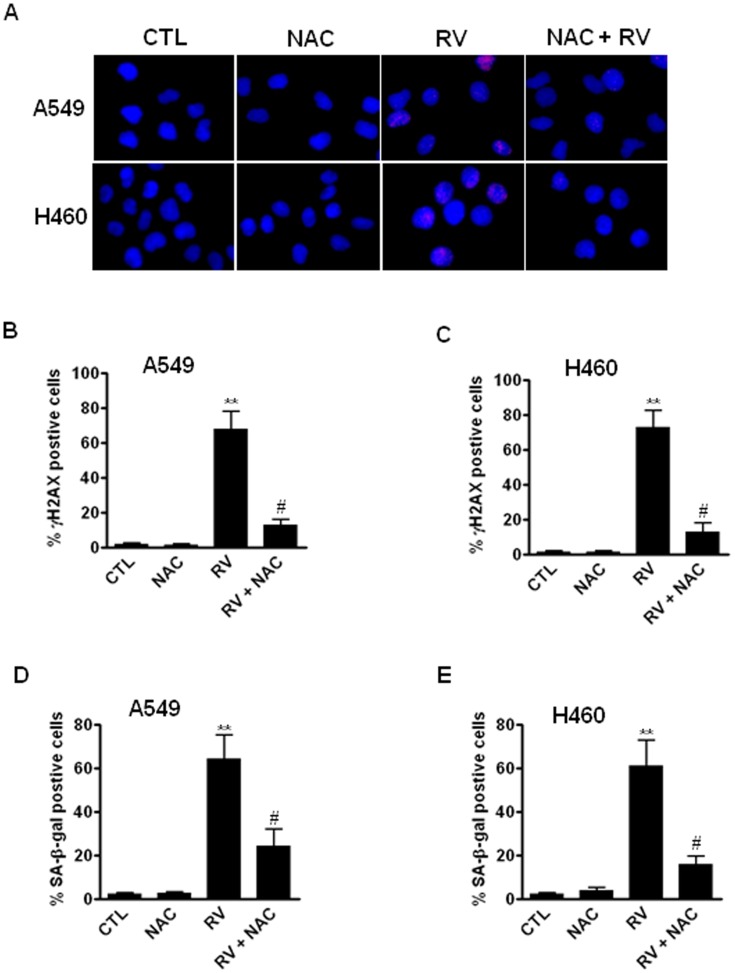
Inhibition of ROS by NAC attenuates RV-induced DNA damage and premature senescence in lung cancer cells. (**A**) DNA DSBs were determined by γH2AX immunofluorescent microscopy as previously described (16). Representative immunofluorescent images of γH2AX foci in A549 and H460 cells are presented. (**B**) Inhibition of ROS by NAC (5 mM) diminishes RV (30 µM)-induced γH2AX foci in A549 cells. (**C**) Inhibition of ROS by NAC (5 mM) diminishes RV (30 µM)-induced γH2AX foci in H460 cells. (**D**) Inhibition of ROS by NAC attenuates RV-induced premature senescence in A549 cells. (**E**) Inhibition of ROS by NAC attenuates RV-induced premature senescence in H460 cells. **, *p*<0.001 vs. control. ^#^, *p*<0.01 vs. RV.

Although our data have shown that RV-induced DNA damage is associated with increased ROS production in NSCLC cells (**Fig. 4**), it has yet to be determined if inhibition of ROS production using antioxidants can prevent RV-induced DNA damage and premature senescence. To this end, we pre-incubated cells with NAC prior to RV treatment to determine if NAC can attenuate RV-induced DNA DSBs and premature senescence in lung cancer cells. As shown in **Figure 5A**, our data demonstrate that pretreatment with NAC significantly inhibits the formation of RV-induced γH2AX foci in A549 and H460 cells. Furthermore, SA-β-gal staining results show that the percentage of RV-induced premature senescent cells is substantially reduced in NAC-treated cells (**Figs. 5D and 5E**). Taken together, these findings strongly support the hypothesis that RV induces lung cancer cell premature senescence via ROS-mediated DNA damage.

### RV induces Nox5 expression in lung cancer cells

Next, we sought to determine the mechanisms by which RV induces ROS generation in cancer cells. It was reported that increased intracellular cyclic AMP (cAMP) may contribute to mitochondrial ROS accumulation [39]. Interestingly, a recent study by Park et al. has shown that RV treatment increases the levels of cAMP in mouse C2C12 cells [40]. To determine if RV alters cAMP levels and in turn induces ROS generation in lung cancer cells, we detected cAMP levels in A549 and H460 cells after different does of RV treatment. The EIA results show that RV treatment has no significant effect on cAMP levels in A549 cells (**Figure S1A**). More interestingly, the data demonstrate that RV inhibits the levels of cAMP in H460 cells (**Figure S1B**). These results suggest that cAMP may not be a key player in mediating RV-induced ROS generation in lung cancer cells.

**Figure 6:**
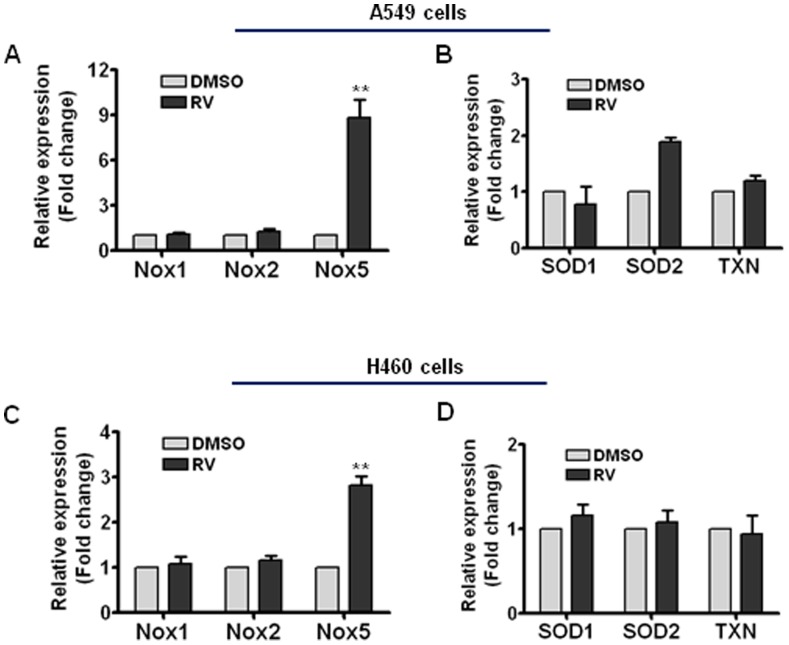
RV induces Nox5 mRNA expression in LSCLC cells. (**A**) Cells were treated with 50 µM of RV or DMSO as vehicle control. Twenty-four hours after RV treatment, the expression levels of Nox1, Nox2, and Nox5 mRNAs were determined using real-time RT-PCR. (**B**) The expression levels of SOD1, SOD2 and TXN in A549 cells were determined by real-time RT-PCR. (**C**) The expression levels of Nox1, Nox2, and Nox5 in H460 cells are presented as fold change (mean ± SEM). (**D**) The expression levels of SOD1, SOD2 and TXN in H460 cells are presented. **, *p*<0.001 vs. control.

The NADPH oxidases (Noxs) are a family of transmembrane enzymes that generate superoxide and other ROS [41]. To better understand how RV induces ROS generation in cancer cells, we investigated if RV treatment has any impact on the expression of Nox1, Nox2, Nox3, Nox4 and Nox5 in NSCLC cells. Real-time RT-PCR results indicate that Nox1, 2 and 5 are abundantly expressed in both A549 and H460 cells, whereas Nox 3 and 4 are barely detectable in lung cancer cells (**Figure S2**). Surprisingly, our data reveal that RV treatment selectively increases Nox5 expression in both A549 and H460 cells (**Figs. 6A and 6C** **)**, suggesting that RV-induced ROS generation in cancer cells is likely attributable to increased Nox5 expression. Given the important roles of antioxidant enzymes such as mitochondrial superoxide dismutase (SOD) and thioredoxin (TXN) in modulating intracellular ROS balance [42], we decided to determine if RV treatment affects the expression of SOD and TXN in lung cancer cells. The real-time PCR data demonstrate that RV treatment only causes a modest increase (less than 2-fold) in SOD2 expression in A549 cells, but has no effect on the expression of SOD1, SOD2 and TXN mRNAs in H460 cells (**Figs. 6B and 6D**). Together, these data suggest that RV may induce ROS generation in cancer cells through up-regulating Nox5 expression.

## Discussion

Cellular senescence is a state of permanent cell cycle arrest that can be triggered by a variety of stresses including DNA damage, telomere shortening and oxidative stress. Senescence limits the life span and proliferative capacity of cells, therefore the induction of senescence is regarded as an important mechanism of cancer prevention [20]–[22]. More importantly, growing evidence has demonstrated that therapy-induced senescence is a critical mechanism of action for many chemotherapeutic agents and radiation treatment [11], [12], [15], [17], [23]. However, the contribution of senescence induction to RV's anticancer and chemopreventive effects has not been well elucidated. Here we provide experimental data demonstrating that low dose RV treatment inhibits the growth of lung cancer cells via an apoptosis-independent mechanism. The results reveal that RV may exert its anticancer and chemopreventive activities via the induction of senescence in cancer cells. Consistent with our observations, Rusin et al. also reported that RV treatment induces senescence-like phenotype in cancer cells [43]. This is a significant finding because the induction of senescence, as opposed to apoptosis, requires much lower concentration of RV, suggesting RV could be useful clinically. Moreover, these studies underscore the importance of senescence induction in mediating RV's chemopreventive and anticancer effects.

It has been well-established that induction of DNA damage is a central mechanism through which many anticancer agents including ionizing radiation kill tumor cells [13], [15], [38], [44]. Of the various types of DNA damage, DNA DSBs are the most cytotoxic because of their great potential to cause cell death and/or cell cycle arrest. Thus, an increased capacity of DNA damage repair in tumor cells was thought to be an important contributor to therapy-resistance during cancer treatments [45]. Interestingly, it has been shown that many DNA-damaging agents such as ionizing radiation can induce DNA damage-mediated premature senescence in cancer cells, suggesting that these therapeutic agents may exert their anticancer effects via DNA damage-mediated premature senescence [13], [15]. Although our data have shown that RV induces premature senescence in lung cancer cells in a dose-dependent manner, it was largely unknown if DNA damage is involved in RV-induced senescence in tumor cells. This study revealed that γH2AX foci, an important surrogate of DNA DSBs, were markedly increased in RV-treated NSCLC cells as compared with control cells. These data suggest that DNA damage-induced premature senescence may contribute to the anticancer effects of RV. Consistent with our observations, it was found that RV can induce plasmid DNA breaks in the presence of Cu (II) and under oxidative conditions [46]. These results support the hypothesis that low dose RV treatment suppresses the growth of lung cancer cells via DNA damage-induced premature senescence.

Activation of the p53–p21 pathway by DNA damage has been shown to be a critical mechanism underlying SIPS [12], [15], [17], [18]. Here we show that RV-induced premature senescence is associated with increased expression of p53 and p21 in NSCLC cells, suggesting that activation of the p53–p21 pathway may play an important role in modulating RV-induced senescence in lung cancer cells. More importantly, it was also found that RV-induced senescence correlates well with a significant decrease in EF1A expression in A549 and H460 cells. These novel findings demonstrate, for the first time, that down-regulation of EF1A is involved in RV-induced premature senescence in lung cancer cells. Consistent with these observations, a recent study has suggested that decreased expression of EF1A is a potential biomarker of premature senescence [47]. However, further studies will be needed to define the exact role of EF1A in modulating RV-induced premature senescence in cancer cells.

Many anticancer agents and ionizing radiation destroy tumor cells largely through the generation of ROS [48]. Moreover, increased ROS can trigger oxidative DNA damage and cause DNA DSBs, thus leading to premature senescence [37]. To determine the role of ROS in RV-induced premature senescence in lung cancer cells, we investigated the levels of ROS in RV-treated A549 and H460 cells using DCF-DA staining and flow cytometric assays. The data show that RV-induced senescence is associated with increased ROS production and DNA DSBs in lung cancer cells, suggesting that RV may induce premature senescence in lung cancer cells via ROS-mediated DNA damage. The important contribution of ROS to RV-induced DNA damage and premature senescence was further confirmed by the observations that inhibition of ROS production by NAC attenuates RV-induced DNA damage and senescence in NSCLC cells. Consistent with these observations, a pro-oxidant effect of RV was also observed in U937 leukemia cells and was characterized by the depletion of GSH and an increase in ROS production [49]. Moreover, previous studies by Hadi and coworkers also showed that RV could increase ROS generation and ROS-induced DNA damage in human peripheral lymphocytes [50], [51]. Together, these findings demonstrate that low dose RV inhibits the growth of lung cancer cells via the induction of senescence through ROS-mediated DNA damage.

It is worth noting that there is evidence that RV can act as an ROS scavenger in normal cells to protect against ionizing radiation-induced oxidative stress and tissue injury [52], suggesting that RV may have differential effects on ROS production in normal versus cancer cells. Given that aberrant redox systems are frequently observed in many tumor cells [48], [53], [54], it is possible that RV may selectively suppress the growth of tumor cells with little or no toxicity to normal cells due to their differential redox status. In agreement with this hypothesis, our data show that RV treatment has no significant effect on the expression of SOD1, SOD2 and TXN in H460 lung cancer cells, although it was reported that RV could induce a substantial (more than 6-fold) increase in SOD2 expression in normal cells [55]. More importantly, our studies demonstrate for the first time that RV selectively increases Nox5 expression in NSCLC cells, suggesting that RV may induce ROS generation in cancer cells via upregulating Nox5 expression.

## Materials and Methods

### Reagents

Resveratrol (Trans-3, 4′, 5-trihydroxystilnene) and all other chemicals were purchased from Sigma (St. Louis, MO). Dulbecco's modified Eagle's medium (DMEM) and other culture media were obtained from Invitrogen (Carlsbad, CA). Rabbit anti-human p53 antibody and rabbit anti-human EF1A monoclonal antibody were purchased from Cell Signaling (Danvers, MA). Mouse anti-human p21 monoclonal antibody was obtained from Santa Cruz Biotechnology. Monoclonal β-actin antibody was purchased from Sigma. A senescence-associated β-galactosidase (SA-β-gal) staining kit was purchased from Cell Signaling. The mouse anti-phospho-histone H2AX (γH2AX) monoclonal antibody was purchased from Millipore (Billerica, MA). TRIzol reagent and SuperScript III first-stand synthesis system were purchased from Invitrogen (Carlsbad, CA). Cyclic AMP (cAMP) EIA kit was purchased from Cayman Chemical (Ann Arbor, MI).

### Cell lines and culture

Human non-small cell lung cancer (NSCLC) cell lines A549 and H460 were purchased from American Type Culture Collection. A549 cells were cultured in DMEM medium containing 10% FBS, 2 mM L-glutamine and 100 microgram/ml of penicillin-streptomycin (Invitrogen). H460 cells were grown in RPMI-1640 medium containing 10% FBS, 2 mM L-glutamine and 100 microgram/ml of penicillin-streptomycin (Invitrogen).

### Clonogenic survival assay

Clonogenic assays were performed to determine the effects of RV treatment on the colony-forming ability of NSCLC cells. Briefly, A549 and H460 lung cancer cells were cultured at low density in the presence of different concentrations of RV or DMSO as vehicle control in 60 mm dishes for 10 to 12 days to allow the formation cell colonies. Colonies were fixed and stained with 0.5% crystal violet (Sigma) in methanol for 30 min. The number of colonies(≥50 cells) was scored using a microscopy.

Senescence-associated β-galactosidase (SA-β-gal) staining

In situ staining of SA-β-gal was performed using a senescence β-galactosidase staining kit (Cell Signaling) as previously described [56].

### Western blotting analysis

A549 and H460 cells were treated with different doses of RV or DMSO as control. Total cell proteins were prepared at 24 h post treatment using cell lysis buffer (Cell Signaling) supplemented with a cocktail of proteinase inhibitors (Sigma). Western blotting analysis was performed as previously described [56]. Briefly, fifty microgram of protein samples were resolved on 10% Mini-Protean TGX gels (Bio-Rad) and transferred onto 0.2 µM PVDF membrane (Millipore). Blots were blocked with 5% non-fat milk for 1–2 hrs at room temperature and then probed with primary antibodies and incubated at 4°C overnight. After extensive washing with TBS-T, blots were incubated with appropriate HRP-conjugated secondary antibody for 1 h at room temperature. Protein bands were detected using an ECL Plus Western Blotting Detection System (GE Healthcare Life Science).

### Immunofluorescent microscopic analysis of γH2AX foci

Cells were cultured on 4-well chamber slides overnight and the next day treated with RV or DMSO (vehicle control). At the end of desired treatments times, cells were fixed with ice-cold 4% paraformaldehyde for 10 min and washed twice with PBS. Then the cells were permeabilized with 0.2% Triton X-100/PBS on ice for 10 min. Slides were blocked with 5% normal goat serum for 30 min before incubation with mouse anti-phospho H2AX (S139) monoclonal antibody for 2 h at room temperature or overnight at 4°C. Cells were incubated with Alexa Fluor 555-conjugated anti-mouse IgG secondary antibody (Invitrogen) for 1 h at room temperature. Nuclei were counterstained with DAPI. Slides were mounted with Vectashield (Vector Laboratories, Burlingame, CA). The γH2AX foci were viewed by a Zeiss Axio Observer Z1, and images were captured using AxioVison 6.4 software (Carl Zeiss, Oberkochen Germany).

### Flow cytometric analysis of ROS

Intracellular ROS were measured by flow cytometric analysis as we have previously reported [37]. Briefly, cells were loaded with 5 µM of 2′, 7′-dichlorodihydrofluorescein diacetate (DCF-DA) and incubated at 37°C for 30 min. The peak excitation wavelength for oxidized DCF-DA was 488 nm and emission was 525 nm.

### Cyclic AMP (cAMP) immunoassay

Cells were pre-incubated for 30 min with 0.5 mM isobutyl methylxanthine (IBMX) and then treated with different doses of RV. At 30 min after RV treatment, the medium was removed and the cells were washed twice with PBS containing 0.5 mM IBMX to inhibit phosphodiesterase and to prevent the breakdown of the cAMP during sample collection and processing. The levels of cAMP in A549 and H460 cells were measured using a cAMP EIA kit (Cayman Chemical) according to the manufacturer's instructions. The application of this assay for cAMP measurement has been well-documented in recent publications [57], [58].

### Real-time reverse transcriptase-PCR (RT-PCR)

Total cellular RNA was prepared using TRIzol reagent (Invitrogen). First-strand cDNA was synthesized from 2 µg of total RNA using SuperScript III first-strand synthesis system (Invitrogen) according to the manufacturer's instructions. The expression levels of SOD1, SOD2, TXN, Nox1, Nox2, Nox3, Nox4 and Nox5 mRNAs were determined by real-time RT-PCR using SYBR Green I Master (Roche) and a Light Cycler 480 system (Roche). The changes in mRNA expression were calculated by the comparative Ct method as described previously [59]. Data were normalized to GAPDH expression. Primer sequences were listed in Table S1.

### Statistical analysis

All experiments were repeated independently at least three times. Paired comparisons were carried out using Student's *t*-test. Multiple group comparisons were performed using analysis of variance (ANOVA). Differences were considered statistically significant at *p*<0.05. All analyses were carried out with the GraphPad Prism program from GraphPad Software, Inc. (San Diego, CA).

## Supporting Information

Figure S1 **Effect of RV on the levels of cAMP in lung cancer cells.** (**A**) The levels of cAMP in A549 cells after different doses of RV treatment were determined using a cAMP EIA kit (Cayman Chemical) according to the manufacturer's instructions. The results are presented as mean ± SEM. (**B**) The levels of cAMP in H460 cells were determined using a cAMP EIA kit and are presented as mean ± SEM. *, *p*<0.05 vs. DMSO control.
(TIF)

Figure S2 **Real-time RT-PCR analysis of Nox1, 2, 3, 4, and 5 expression in lung cancer cells.** (**A**) Relative expression levels of Nox1, 2, 3, 4, and 5 mRNAs in A549 cells were determined by real-time RT-PCR and are presented as mean delta Ct ± SEM. (**B**) Relative expression levels of Nox1, 2, 3, 4, and 5 mRNAs in H460 cells are presented as mean delta Ct ± SEM.
(TIF)

Table S1 **Sequences of real-time PCR primers used for this study.**
(DOCX)

## References

[1] Jemal A, Siegel R, Ward E, Hao Y, Xu J, et al (2009) Cancer statistics 2009. CA Cancer J Clin 59: 225–49. 19474385 10.3322/caac.20006

[2] Minna JD, Roth JA, Gazdar AF (2002) Focus on lung cancer. Cancer Cell 1: 49–52. 12086887 10.1016/s1535-6108(02)00027-2

[3] Gullett NP, Ruhul Amin AR, Bayraktar S, Pezzuto JM, Shin DM, et al (2010) Cancer prevention with natural compounds. Semin Oncol 37: 258–81. 20709209 10.1053/j.seminoncol.2010.06.014

[4] Rajamanickam S, Agarwal R (2008) Natural products and colon cancer: current status and future prospects. Drug Dev Res 69: 460–471. 19884979 10.1002/ddr.20276 PMC2659299

[5] Cottart CH, Nivet-Antoine V, Laguillier-Morizot C, Beaudeux JL (2010) Resveratrol bioavailability and toxicity in humans. Mol Nutr Food Res 54: 7–16. 20013887 10.1002/mnfr.200900437

[6] Patel KR, Brown VA, Jones DJ, Britton RG, Hemingway D, et al (2010) Clinical pharmacology of resveratrol and its metabolites in colorectal cancer patients. Cancer Res 70: 7392–9. 20841478 10.1158/0008-5472.CAN-10-2027 PMC2948608

[7] Patel KR, Scott E, Brown VA, Gescher AJ, Steward WP, et al (2011) Clinical trials of resveratrol. Ann N Y Acad Sci 1215: 161–9. 21261655 10.1111/j.1749-6632.2010.05853.x

[8] Gupta SC, Kannappan R, Reuter S, Kim JH, Aggarwal BB (2011) Chemosensitization of tumors by resveratrol. Ann N Y Acad Sci 1215: 150–60. 21261654 10.1111/j.1749-6632.2010.05852.x PMC3060406

[9] Baur JA, Sinclair DA (2006) Therapeutic potential of resveratrol: the in vivo evidence. Nat Rev Drug Discov 5: 493–506. 16732220 10.1038/nrd2060

[10] Bhardwaj A, Sethi G, Vadhan-Raj S, Bueso-Ramos C, Takada Y, et al (2007) Resveratrol inhibits proliferation, induces apoptosis, and overcomes chemoresistance through down-regulation of STAT3 and nuclear factor-kappaB-regulated antiapoptotic and cell survival gene products in human multiple myeloma cells. Blood 109: 2293–302. 17164350 10.1182/blood-2006-02-003988

[11] Gewirtz DA, Holt SE, Elmore LW (2008) Accelerated senescence: an emerging role in tumor cell response to chemotherapy and radiation. Biochem Pharmacol 76: 947–57. 18657518 10.1016/j.bcp.2008.06.024

[12] Ewald JA, Desotelle JA, Wilding G, Jarrard DF (2010) Therapy-induced senescence in cancer. J Natl Cancer Inst 102: 1536–46. 20858887 10.1093/jnci/djq364 PMC2957429

[13] te Poele RH, Okorokov AL, Jardine L, Cummings J, Joel SP (2002) DNA damage is able to induce senescence in tumor cells in vitro and in vivo. Cancer Res 62: 1876–83. 11912168

[14] Hayflick L, Moorhead PS (1961) The serial cultivation of human diploid cell strains. Exp Cell Res 25: 585–621. 13905658 10.1016/0014-4827(61)90192-6

[15] Jones KR, Elmore LW, Jackson-Cook C, Demasters G, Povirk LF, et al (2005) p53-dependent accelerated senescence induced by ionizing radiation in breast tumor cells. Int J Radiat Biol 81: 445–58. 16308915 10.1080/09553000500168549

[16] Dimri GP, Lee X, Basile G, Acosta M, Scott G, et al (1995) A biomarker that identifies senescent human cells in culture and in aging skin in vivo. Proc Natl Acad Sci USA 92: 9363–7. 7568133 10.1073/pnas.92.20.9363 PMC40985

[17] Chang BD, Swift ME, Shen M, Fang J, Broude EV, et al (2002) Molecular determinants of terminal growth arrest induced in tumor cells by a chemotherapeutic agent. Proc Natl Acad Sci U S A 99: 389–94. 11752408 10.1073/pnas.012602599 PMC117570

[18] Chen QM, Prowse KR, Tu VC, Purdom S, Linskens MH (2001) Uncoupling the senescent phenotype from telomere shortening in hydrogen peroxide-treated fibroblasts. Exp Cell Res 265: 294–303. 11302695 10.1006/excr.2001.5182

[19] Chen JH, Hales CN, Ozanne SE (2007) DNA damage, cellular senescence and organismal ageing: causal or correlative? Nucleic Acids Res 35: 7417–28. 17913751 10.1093/nar/gkm681 PMC2190714

[20] Braig M, Lee S, Loddenkemper C, Rudolph C, Peters AH, et al (2005) Oncogene-induced senescence as an initial barrier in lymphoma development. Nature 436: 660–5. 16079837 10.1038/nature03841

[21] Guo X, Keyes WM, Papazoglu C, Zuber J, Li W, et al (2009) TAp63 induces senescence and suppresses tumorigenesis in vivo. Nat Cell Biol 11: 1451–7. 19898465 10.1038/ncb1988 PMC2920298

[22] Bennecke M, Kriegl L, Bajbouj M, Retzlaff K, Robine S, et al (2010) Ink4a/Arf and oncogene-induced senescence prevent tumor progression during alternative colorectal tumorigenesis. Cancer Cell 18: 135–46. 20708155 10.1016/j.ccr.2010.06.013

[23] Schmitt CA, Fridman JS, Yang M, Lee S, Baranov E, et al (2002) A senescence program controlled by p53 and p16INK4a contributes to the outcome of cancer therapy. Cell 109: 335–46. 12015983 10.1016/s0092-8674(02)00734-1

[24] Hussain AR, Uddin S, Bu R, Khan OS, Ahmed SO, et al (2011) Resveratrol suppresses constitutive activation of AKT via generation of ROS and induces apoptosis in diffuse large B cell lymphoma cell lines. PLoS One 6: e24703. 21931821 10.1371/journal.pone.0024703 PMC3171480

[25] Bhat KP, Lantvit D, Christov K, Mehta RG, Moon RC, et al (2001) Estrogenic and antiestrogenic properties of resveratrol in mammary tumor models. Cancer Res 61: 7456–63. 11606380

[26] Sale S, Tunstall RG, Ruparelia KC, Potter GA, Steward WP, et al (2005) Comparison of the effects of the chemopreventive agent resveratrol and its synthetic analog trans 3,4,5,4′-tetramethoxystilbene (DMU-212) on adenoma development in the Apc(Min+) mouse and cyclooxygenase-2 in human-derived colon cancer cells. Int J Cancer 115: 194–201. 15688382 10.1002/ijc.20884

[27] Harikumar KB, Kunnumakkara AB, Sethi G, Diagaradjane P, Anand P, et al (2010) Resveratrol, a multitargeted agent, can enhance antitumor activity of gemcitabine in vitro and in orthotopic mouse model of human pancreatic cancer. Int J Cancer 127: 257–68. 19908231 10.1002/ijc.25041 PMC3090706

[28] Whitlock NC, Bahn JH, Lee SH, Eling TE, Baek SJ (2011) Resveratrol-induced apoptosis is mediated by early growth response-1, Krüppel-like factor 4, and activating transcription factor 3. Cancer Prev Res (Phila) 4: 116–27. 21205742 10.1158/1940-6207.CAPR-10-0218 PMC3064282

[29] Kao CL, Huang PI, Tsai PH, Tsai ML, Lo JF, et al (2009) Resveratrol-induced apoptosis and increased radiosensitivity in CD133-positive cells derived from atypical teratoid/rhabdoid tumor. Int J Radiat Oncol Biol Phys 74: 219–28. 19362240 10.1016/j.ijrobp.2008.12.035

[30] Cui J, Sun R, Yu Y, Gou S, Zhao G, et al (2010) Antiproliferative effect of resveratrol in pancreatic cancer cells. Phytother Res 24: 1637–44. 21031621 10.1002/ptr.3157

[31] Jiang H, Zhang L, Kuo J, Kuo K, Gautam SC, et al (2005) Resveratrol-induced apoptotic death in human U251 glioma cells. Mol Cancer Ther 4: 554–61. 15827328 10.1158/1535-7163.MCT-04-0056

[32] Scott E, Steward WP, Gescher AJ, Brown K (2012) Resveratrol in human cancer chemoprevention--choosing the 'right' dose. Mol Nutr Food Res 56: 7–13. 22218912 10.1002/mnfr.201100400

[33] Lazebnik YA, Kaufmann SH, Desnoyers S, Poirier GG, Earnshaw WC (1994) Cleavage of poly (ADP-ribose) polymerase by a proteinase with properties like ICE. Nature 371: 346–7. 8090205 10.1038/371346a0

[34] Nicholson DW, Ali A, Thornberry NA, Vaillancourt JP, Ding CK, et al (1995) Identification and inhibition of the ICE/CED-3 protease necessary for mammalian apoptosis. Nature 376: 37–43. 7596430 10.1038/376037a0

[35] Ben-Porath I, Weinberg RA (2004) When cells get stressed: an integrative view of cellular senescence. J Clin Invest 113: 8–13. 14702100 10.1172/JCI200420663 PMC300889

[36] Rothkamm K, Löbrich M (2003) Evidence for a lack of DNA double-strand break repair in human cells exposed to very low x-ray doses. Proc Natl Acad Sci U S A 100: 5057–62. 12679524 10.1073/pnas.0830918100 PMC154297

[37] Wang Y, Liu L, Pazhanisamy SP, Meng A, Zhou D (2010) Total body irradiation selectively induces persistent oxidative stress in murine hematopoietic stem cells. Free Radic Biol and Med 48: 348–56. 19925862 10.1016/j.freeradbiomed.2009.11.005 PMC2818724

[38] Huang HL, Fang LW, Lu SP, Chou CK, Luh TY, et al (2003) DNA-damaging reagents induce apoptosis through reactive oxygen species-dependent Fas aggregation. Oncogene 22: 8168–77. 14603257 10.1038/sj.onc.1206979

[39] Zhang L, Seitz LC, Abramczyk AM, Chan C (2010) Synergistic effect of cAMP and palmitate in promoting altered mitochondrial function and cell death in HepG2 cells. Exp Cell Res 316: 716–27. 20026039 10.1016/j.yexcr.2009.12.008 PMC2826503

[40] Park SJ, Ahmad F, Philp A, Baar K, Williams T, et al (2012) Resveratrol ameliorates aging-related metabolic phenotypes by inhibiting cAMP phosphodiesterases. Cell 148: 421–33. 22304913 10.1016/j.cell.2012.01.017 PMC3431801

[41] Bedard K, Jaquet V, Krause KH (2012) NOX5: from basic biology to signaling and disease. Free Radic Biol Med 52: 725–34. 22182486 10.1016/j.freeradbiomed.2011.11.023

[42] Kim A, Joseph S, Khan A, Epstein CJ, Sobel R, et al (2010) Enhanced expression of mitochondrial superoxide dismutase leads to prolonged in vivo cell cycle progression and up-regulation of mitochondrial thioredoxin. Free Radic Biol Med 48(11): 1501–12. 20188820 10.1016/j.freeradbiomed.2010.02.028 PMC2945707

[43] Rusin M, Zajkowicz A, Butkiewicz D (2009) Resveratrol induces senescence-like growth inhibition of U-2 OS cells associated with the instability of telomeric DNA and upregulation of BRCA1. Mech Ageing Dev 130: 528–37. 19559722 10.1016/j.mad.2009.06.005

[44] Cohen SM, Lippard SJ (2001) Cisplatin: from DNA damage to cancer chemotherapy. Prog Nucleic Acid Res Mol Biol 67: 93–130. 11525387 10.1016/s0079-6603(01)67026-0

[45] Tobin LA, Robert C, Nagaria P, Chumsri S, Twaddell W, et al (2012) Targeting abnormal DNA repair in therapy-resistant breast cancers. Mol Cancer Res 10: 96–107. 22112941 10.1158/1541-7786.MCR-11-0255 PMC3319138

[46] Fukuhara K, Nagakawa M, Nakanishi I, Ohkubo K, Imai K, et al (2006) Structural basis for DNA-cleaving activity of resveratrol in the presence of Cu(II). Bioorg Med Chem 14: 1437–43. 16249091 10.1016/j.bmc.2005.09.070

[47] Byun HO, Han NK, Lee HJ, Kim KB, Ko YG, et al (2009) Cathepsin D and eukaryotic translation elongation factor 1 as promising markers of cellular senescence. Cancer Res 69: 4638–47. 19487283 10.1158/0008-5472.CAN-08-4042

[48] Wondrak GT (2009) Redox-Directed Cancer Therapeutics: Molecular Mechanisms and Opportunities. Antioxid Redox Signal 11: 3013–3069. 19496700 10.1089/ars.2009.2541 PMC2824519

[49] Guha P, Dey A, Sen R, Chatterjee M, Chattopadhyay S, et al (2011) Intracellular GSH depletion triggered mitochondrial Bax translocation to accomplish resveratrol-induced apoptosis in the U937 cell line. J Pharmacol Exp Ther 336: 206–14. 20876229 10.1124/jpet.110.171983

[50] Azmi AS, Bhat SH, Hanif S, Hadi SM (2006) Plant polyphenols mobilize endogenous copper in human peripheral lymphocytes leading to oxidative DNA breakage: a putative mechanism for anticancer properties. FEBS Lett 580: 533–8. 16412432 10.1016/j.febslet.2005.12.059

[51] Azmi AS, Bhat SH, Hadi SM (2005) Resveratrol-Cu (II) induced DNA breakage in human peripheral lymphocytes: implications for anticancer properties. FEBS Lett 579: 3131–5. 15919081 10.1016/j.febslet.2005.04.077

[52] Velioğlu-Oğünç A, Sehirli O, Toklu HZ, Ozyurt H, Mayadağli A, et al (2009) Resveratrol protects against irradiation-induced hepatic and ileal damage via its anti-oxidative activity. Free Radic Res 43: 1060–71. 19707923 10.1080/10715760903171100

[53] Toyokuni S, Okamoto K, Yodoi J, Hiai H (1995) Persistent oxidative stress in cancer. FEBS Lett 358: 1–3. 7821417 10.1016/0014-5793(94)01368-b

[54] Szatrowski TP, Nathan CF (1991) Production of large amounts of hydrogen peroxide by human tumor cells. Cancer Res 51: 794–798. 1846317

[55] Robb EL, Page MM, Wiens BE, Stuart JA (2008) Molecular mechanisms of oxidative stress resistance induced by resveratrol: Specific and progressive induction of MnSOD. Biochem Biophys Res Commun 367: 406–12. 18167310 10.1016/j.bbrc.2007.12.138

[56] Wang Y, Meng A, Zhou D (2004) Inhibition of phosphatidylinostol 3-kinase uncouples H2O2-induced senescent phenotype and cell cycle arrest in normal human diploid fibroblasts. Exp Cell Res 298: 188–96. 15242773 10.1016/j.yexcr.2004.04.012

[57] Leopold JA, Dam A, Maron BA, Scribner AW, Liao R, et al (2007) Aldosterone impairs vascular reactivity by decreasing glucose-6-phosphate dehydrogenase activity. Nat Med 13: 189–97. 17273168 10.1038/nm1545 PMC3648863

[58] Zhu W, Jia Q, Wang Y, Zhang Y, Xia M (2012) The anthocyanin cyanidin-3-O-β-glucoside, a flavonoid, increases hepatic glutathione synthesis and protects hepatocytes against reactive oxygen species during hyperglycemia: Involvement of a cAMP-PKA-dependent signaling pathway. Free Radic Biol Med 52: 314–27. 22085656 10.1016/j.freeradbiomed.2011.10.483

[59] Wang Y, Schulte BA, Larue AC, Ogawa M, Zhou D (2006) Total body irradiation selectively induces murine hematopoietic stem cell senescence. Blood 107: 358–66. 16150936 10.1182/blood-2005-04-1418 PMC1895367

