# Supplementary figures and images for: Resveratrol Induces Premature Senescence in Lung Cancer Cells via ROS-Mediated DNA Damage
Source: PLoS One. 2013 Mar 22;8(3):e60065. doi: 10.1371/journal.pone.0060065 (PMC3606183; doi:10.1371/journal.pone.0060065)

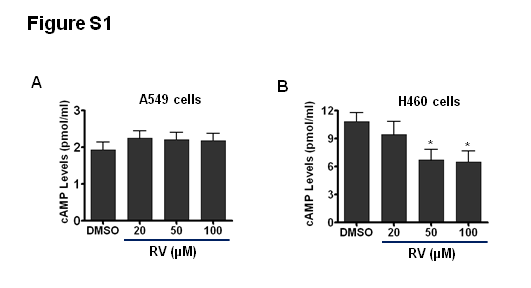

Supplement: Figure S1 — Effect of RV on the levels of cAMP in lung cancer cells. (A) The levels of cAMP in A549 cells after different doses of RV treatment were determined using a cAMP EIA kit (Cayman Chemical) according to the manufacturer's instructions. The results are presented as mean ± SEM. (B) The levels of cAMP in H460 cells were determined using a cAMP EIA kit and are presented as mean ± SEM. *, p<0.05 vs. DMSO control. (TIF) [file pone.0060065.s001.tif]
