# Supplementary material for: Resveratrol Induces Premature Senescence in Lung Cancer Cells via ROS-Mediated DNA Damage
Source: PLoS One. 2013 Mar 22;8(3):e60065. doi: 10.1371/journal.pone.0060065 (PMC3606183; doi:10.1371/journal.pone.0060065)
Supplement: Table S1 — Sequences of real-time PCR primers used for this study. (DOCX) [file pone.0060065.s003.docx]

**Table S1. Real-time RT-PCR primers used for this study**

| Gene name | Access No. | Forward primer | Reverse primer |
| --- | --- | --- | --- |
| Nox1 | NM_013955 | GCAAATGCTGTCACCGATATTC | TGCAGATTACCGTCCTTATTCC |
| Nox2 | NM_000397 | GCTATGAGGTGGTGATGTTAGT | CTTCAGATTGGTGGCGTTATTG |
| Nox3 | NM_015718 | TGAGGGTCTCTCCACCATATT | ACTCCTCCTCTTCATACCAGTAG |
| Nox4 | NM_016931 | ACCTCAACTGCAGCCTTATC | ATCCAACAATCTCCTGGTTCTC |
| Nox5 | NM_024505 | CATCCAGTTCCACCAGCTTAT | AGCCTGGAGTACAAAGTTCAC |
| SOD1 | NM_000454 | TGGAAGTCGTTTGGCTTGT | CAGCTAGCAGGATAACAGATGAG |
| SOD2 | NM_000636 | GGGATGCCTTTCTAGTCCTATTC | TATAGAAAGCCGAGTGTTTCCC |
| TXN | NM_001244938 | GAAGCTCTGTTTGGTGCTTTG | CTCGATCTGCTTCACCATCTT |
